# Rapid bupropion-induced hepatotoxicity: a case report and review of the literature

**DOI:** 10.1186/s13256-018-1563-9

**Published:** 2018-02-24

**Authors:** Sulakchanan Anandabaskaran, Vincent Ho

**Affiliations:** 1 Department of Medicine, Campbelltown Public Hospital, Campbelltown, NSW 2560 Australia; 2 Department of Gastroenterology, Campbelltown Public Hospital, Campbelltown, NSW 2560 Australia

**Keywords:** Bupropion, Adverse effects, Hepatotoxicity, Management

## Abstract

**Background:**

Bupropion is an antidepressant that is also used as a non-nicotine method to aid in smoking cessation. Bupropion-induced hepatotoxicity is quoted to affect between 0.1% and 1% of treated patients with either a hepatocellular and/or cholestatic pattern of damage. The mechanism of damage is considered to be predominantly immune-mediated with the presence of a hypersensitivity syndrome (fever, rash, eosinophilia, autoantibodies) and a short latency period (1–6 weeks). We believe our reporting of this case to the already existing small list of only seven cases in the world literature will help practicing physicians to deal with the diagnostic and management dilemmas that bupropion-induced hepatotoxicity brings.

**Case presentation:**

A 50-year-old Caucasian woman presented to our hospital with significant derangement of liver transaminases after 6 days of bupropion treatment for smoking cessation. The patient’s other medications were considered unlikely to be the cause of the hepatotoxicity and were therefore continued. The patient’s liver function tests normalized on withdrawal of bupropion, confirming that bupropion was the probable cause of the patient’s hepatotoxicity.

**Conclusions:**

We conclude that hepatotoxicity is a rare adverse effect of bupropion use, but physicians should be aware of the possibility of this potentially serious clinical picture of drug-induced hepatotoxicity with varied clinical presentation and prognosis.

## Background

Medications are a common cause of liver injury because the liver is the main site of drug clearance, biotransformation, and elimination. Bupropion is a commonly used medication to aid with smoking cessation and was also widely used in the past as an antidepressant. The most common adverse effects are dry mouth, headache, insomnia, and agitation. Drug-induced hepatotoxicity, however, is a very rare adverse event, with only seven cases reported in the world literature. Having reviewed these cases, we note that the presentation, mortality, and treatment varied in each of these cases, creating both diagnostic and management dilemmas. We report a patient who experienced significant derangement of her liver tests with a clinical vignette highly suggestive of bupropion-induced hepatotoxicity. We believe further additions to the already existing small literature base on this topic will help practicing physicians when dealing with the possibility of bupropion-induced hepatotoxicity.

## Case presentation

A 50-year-old Caucasian woman with a 6-month history of Crohn’s disease and receiving methotrexate for this disease presented with deranged liver function tests to our gastroenterology clinic. She had recently been discharged from the hospital 1 week earlier, following a flare of her Crohn’s disease. On discharge, she was sent home with nasogastric feeding to help with her malnutrition, and as part of recognizing any refeeding syndrome, she was having regular blood tests in the community. Her routine blood tests on 19 December 2016 showed marked derangement in her transaminases, with aspartate transaminase (AST) of 787 U/L and alanine transaminase (ALT) of 1032 U/L. Her bilirubin and alkaline phosphatase (ALP) were normal, and her γ-glutamyl transferase (GGT) was only mildly raised at 51 U/L. Results of her liver tests done 3 days prior, on 16 December, following discharge from the hospital were completely normal. Of note, she was started on 150 mg of bupropion on 13 December, which was increased to 150 mg twice daily 3 days later, on 16 December, to assist with smoking cessation. Results of her remaining blood tests, apart from long-standing stable normocytic anemia, were unremarkable.

The patient had a history of osteoporosis, palpitations, and depression. Her regular medications for these diagnoses included methotrexate 15 mg weekly for Crohn’s colitis, folic acid 5 mg weekly, cholecalciferol, sertraline, melatonin, propranolol, conjugated estrogen (Premarin; Wyeth Pharmaceuticals, Philadelphia, PA, USA) as hormone replacement therapy for menopause, pantoprazole, oxycodone, Coloxyl (Aspen Australia, St. Leonard’s, Australia), and a 7-week weaning course of prednisone (from 35 mg daily) for her recent Crohn’s flare with co-trimoxazole cover to continue until weaned off prednisone.

**Fig. 1:**
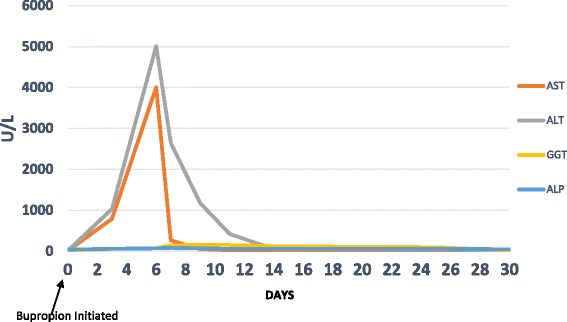
Trends of the patient’s liver function tests. *ALP* Alkaline phosphatase, *ALT* Alanine transaminase, *AST* Aspartate transaminase, *GGT* γ-Glutamyl transferase

The patient’s blood tests on 20 December, done following the initial derangement on 19 December, were even more markedly deranged, with AST of 4006 U/L and ALT of 5007 U/L. Her GGT was also mildly more raised at 73 U/L. However, her bilirubin and ALP remained normal. She was reviewed on 22 December in the gastroenterology clinic. She had weaned to 30 mg of prednisone at that point. There had been no new medications other than the bupropion added since her discharge from the hospital. On examination, she did not have any jaundice, bruising, or any signs of chronic liver disease. Because she was feeling subjectively well with a normal bilirubin level, she was managed in the community setting. Her bupropion had already been stopped on 20 December by her general practitioner. All her usual medications, including prednisone and co-trimoxazole, were continued. At her 22 December clinical review, apart from an increase in GGT to 138 U/L, the remaining markers showed significant improvement with the AST decreased to 263 U/L and ALT reduced to 2643 U/L. Hence, a liver biopsy was not pursued, and instead she was followed with further surveillance liver tests, which continued to show improvement with subsequent near-normalization almost 4 weeks later, on 12 January, with ALT of 39 U/L and GGT of 94 U/L. Her AST had already normalized by 27 December. The trends of her liver tests are shown in Fig. 1.

## Discussion

Bupropion is an antidepressant that is also used as a nonnicotine method to aid in smoking cessation, and it acts by inhibiting the reuptake of dopamine and noradrenaline (norepinephrine) [1]. Preclinical animal toxicology studies have demonstrated reversible hepatotoxicity, hepatocellular hypertrophy, and focal hepatic hyperplasia related to hepatic enzyme induction [2]. Bupropion-induced hepatotoxicity is quoted to affect between 0.1% and 1% of treated patients with a hepatocellular and/or a cholestatic pattern of damage [3]. The mechanism of damage is considered to be predominantly immune-mediated with the presence of a hypersensitivity syndrome (fever, rash, eosinophilia, autoantibodies) and a short latency period (1–6 weeks) [3]. Looking through the literature, we found seven reported cases of hepatotoxicity considered to be induced by bupropion and one further case of hepatotoxicity due to conjoint therapy with bupropion and doxycycline. The features of these cases are summarized in Table 1.

**Table 1:** Bupropion-induced hepatotoxicity case reports in the literature

First author, year [reference]	Dose	Duration of use (days)	Peak ALT/AST (U/L)	Peak bilirubin (mg/dl)	ANA	Outcome
Oslin, 1993 [11]	300 mg daily × 21 days, then 400 mg daily	54	5.4 × ULN/7.8 × ULN	Not recorded	Not recorded	Complete resolution within 10 days of withdrawal of bupropion without needing steroids
Hu, 2000 [8]	200 mg daily	42	6660/9040	3.8 (Normal)	Negative	Complete resolution within 10 days of withdrawal of bupropion without needing steroids
Alvaro, 2001 [9]	150 mg twice daily	20	49 × ULN/68 × ULN	38 (Raised)	Positive (1:80)	Complete resolution 50 days following withdrawal of bupropion, including 20 days of treatment with methylprednisolone (8 mg three times daily)
Khoo, 2003 [12]	150 mg daily	10	674/429	84 (Raised)	Not recorded	Death 19 days after presentation with fulminant liver failure and DIC
Humayun, 2007 [10]	150 mg twice daily	180	1459/1466	37 (Raised)	Positive (1:160)	Death owing to relapse despite initial improvement with prednisone (60 mg/day)
Titos-Arcos, 2008 [13]	150 mg daily × 15 days, then twice daily	30	1405/473	12.14 (Raised)	Positive (1:80)	Complete resolution at 77 days after withdrawal of bupropion without needing steroids
Alonso Rodríguez, 2010 [4]	150 mg daily	Single dose	1116/1837	8.7 (Normal)	Negative	Complete resolution at 20 days after cessation of bupropion without needing steroids
Tang, 2015 [14]	150 mg daily bupropion and 100 mg twice daily doxycycline	14	896/1228	19.9 (Raised)	Negative but SMA-positive	Complete resolution after 67 days with prednisone tapering course starting at 40 mg/day
Our patient	150 mg daily × 3 days, then 150 mg twice daily × 3 days	6	5007/4006	9 μmol/L (Normal)	Not recorded	Near-normalization within 23 days; was already on a weekly tapering course of prednisone for Crohn’s disease flare (30 mg/day at time of initial liver derangement)

*Abbreviations: ALT* Alanine aminotransferase, *AST* Aspartate aminotransferase, *ULN* Upper limit of normal, *ANA* Antinuclear antibody, *SMA* Smooth muscle antibody, *DIC* Disseminated intravascular coagulation

After the Alonso Rodríguez *et al*. [4] case, our patient’s case involved the next shortest time frame (6 days) from initiation of bupropion therapy to the onset of liver enzyme derangement. Given the very small number of cases, owing to the idiosyncratic nature of the drug reaction, there appears to be a large variation in the time frames for the onset of liver derangement after the start of treatment. It is possible that there was an add-on effect from the 5 months of methotrexate use because chronic low-dose methotrexate use has been associated with hepatotoxicity on a histological level, even despite unremarkable liver function tests [5]. However, this is usually expected only with cumulative doses of 1.5 g and above [5], which our patient had not reached (approximately 300 mg in total) by the time of onset of her liver derangement. She was also receiving long-term sertraline and propranolol, which can cause mild liver test abnormalities, including elevations in serum aminotransferase levels in approximately 1–2% of cases [6]; however, the fact that she improved despite continuation of these and her completely normal liver function tests prior to initiation of bupropion make these being partly responsible less likely. The other medication of possibility was the co-trimoxazole started during her hospital admission approximately 2 weeks prior to the liver derangement. Again, the co-trimoxazole cover was continued throughout the subsequent improvement of her liver function tests, which makes it an unlikely cause. The damage with co-trimoxazole is typically cholestatic or mixed with jaundice rather than being a solely hepatocellular picture of liver derangement, as was the case in our patient [7].

Our patient had a good recovery following the initial insult without requiring inpatient admission and with no decompensation of her liver. Using “Hy’s rule” [7], the lack of bilirubinemia did suggest a good prognosis in this case. Our patient subsequently had rapid resolution of her liver function tests, which showed significant improvement within 3–5 days of cessation of bupropion therapy. It is difficult to tell whether the ongoing moderate-dose prednisone therapy for her recent Crohn’s flare had helped in her recovery; however, she had been receiving 30 mg daily of prednisone in the week prior to the derangement of her liver tests and 35 mg daily 2 weeks prior. An antinuclear antibody (ANA) measurement would have been useful to know if there were autoimmune features of her liver derangement and hence the utility of steroid therapy, but this was not carried out; however, there is a possibility of a false-negative result in this case, given the concomitant prednisone therapy for 2 weeks prior to the insult. A liver biopsy was also not carried out in our patient, owing to her rapid improvement after cessation of bupropion. Previous cases in the literature published in 2000 [8], 2001 [9], and 2007 [10] demonstrated liver biopsies in bupropion-induced hepatotoxicity to involve eosinophilic inflammatory infiltration of the portal tracts along with neutrophils and lymphocytes. However, in the case reported by Hu *et al*. [8], there was complete resolution without the use of steroids, but the ANA was negative. In contrast, Alvaro *et al*. [9] and Humayun *et al*. [10] did use steroid treatment, and both cases also had a positive ANA at onset. Interestingly, in the case of Humayun *et al*. [10], there was relapse following withdrawal of steroids after 6 weeks of treatment, with subsequent death occurring despite reinitiation of steroids.

## Conclusions

In summary, given the rapid onset following initiation of bupropion and fast resolution following its cessation, we strongly believe our patient’s case was one of bupropion-induced hepatotoxicity. Bupropion is an effective and safe treatment option for smoking cessation. However, as with many other medications in clinical practice, it does pose a risk of very rare idiosyncratic reactions of hepatotoxicity. The presentation and the clinical outcome following such insult to the liver is varied with bupropion, and management, apart from immediate cessation of the drug, remains unclear, given the very small number of cases in the literature. Steroid treatment is not clearly proven, although there may be a role for steroids in cases of positive of ANA and/or SMA. Owing to the potential for rare but fatal outcomes of bupropion hepatotoxicity, practicing physicians should be aware of this adverse reaction when assessing patients receiving bupropion therapy.
